# T1 hypointense brain lesions in NMOSD and its relevance with disability: a single institution cross-sectional study

**DOI:** 10.1186/s12883-024-03550-1

**Published:** 2024-02-12

**Authors:** Mohamad Ghazanfari Hashemi, Vahid Talebi, Naghmeh Abbasi Kasbi, Mehrshad Abbasi, Nasrin Asgari, Mohammad Ali Sahraian

**Affiliations:** 1 Multiple Sclerosis Research Center, Neuroscience Institute, Tehran University of Medical Sciences, Neurology Department, Sina Hospital, Tehran, Iran; 2 Department of Nuclear Medicine, Vali-Asr Hospital, Tehran University of Medical Sciences, Tehran, Iran; 3 Department of Neurology, Institutes of Regional Health Research and Molecular Medicine, University of Southern Denmark, Odense, Denmark

**Keywords:** Neuromyelitis Optica Spectrum Disorder, Brain MRI, T1 Hypointense Brain Lesion, EDSS

## Abstract

**Background:**

T1 hypointense lesions are considered a surrogate marker of tissue destruction. Although there is a shortage of evidence about T1 hypointense brain lesions, black holes, in patients with Neuromyelitis Optica Spectrum Disorder (NMOSD), the clinical significance of these lesions is not well determined.

**Objectives:**

The impact of T1 hypointense brain lesions on the clinical status and the disability level of patients with NMOSD was sought in this study.

**Methods:**

A total of 83 patients with the final diagnosis of NMOSD were recruited.

Aquaporin-4 measures were collected. The expanded disability status scale (EDSS) and MRI studies were also extracted.

T1 hypointense and T2/FLAIR hyperintense lesions were investigated. The correlation of MRI findings, AQP-4, and EDSS was assessed.

**Results:**

T1 hypointense brain lesions were detected in 22 patients. Mean ± SD EDSS was 3.7 ± 1.5 and significantly higher in patients with brain T1 hypointense lesions than those without them (*p*-value = 0.01). Noticeably, patients with more than four T1 hypointense lesions had EDSS scores ≥ 4. The presence of T2/FLAIR hyperintense brain lesions correlated with EDSS (3.6 ± 1.6 vs 2.3 ± 1.7; *p*-value = 0.01). EDSS was similar between those with and without positive AQP-4 (2.7 ± 1.6 vs. 3.2 ± 1.7; *p*-value = 0.17). Also, positive AQP-4 was not more prevalent in patients with T1 hypointense brain lesions than those without them (50.9 vs 45.4%; *p*-value = 0.8).

**Conclusion:**

We demonstrated that the presence of the brain T1-hypointense lesions corresponds to a higher disability level in NMOSD.

## Introduction

Neuromyelitis optica spectrum disorder (NMOSD) is an autoimmune inflammatory disease of the central nervous system that primarily involves the optic nerves, the spinal cord, and the brainstem [1–3]. The relevance of magnetic resonance imaging (MRI) in NMOSD has been documented for the diagnosis [4] but not for the prediction of the clinical outcome robustly [5]. There is evidence that patients with NMOSD present brain lesions when evaluated by the brain MRI [6–8]. Aside from the necessary role of spinal MRI for diagnosing NMOSD [9], there is an upward trend in the implementation of brain MRI for a precise diagnosis [10].

Brain T1 hypointense lesions or “Black Holes (BH)” are considered one of the imaging characteristics of irreversible tissue destruction in the inflammatory diseases of the CNS, especially Multiple Sclerosis (MS), which is the main differential diagnosis of NMOSD [11]. These lesions harbor a high risk for progression, disability, and poor clinical outcome in patients with MS [12]; however, research addressing T1 hypointense lesions in NMOSD is scarce [13]. Accordingly, we speculate the presence of the brain T1 hypointense lesions may provide prognostic information for patients with NMOSD.

The current study reported the presence of T2/FLAIR hyperintense brain and spinal cord lesions and T1 hypointense lesions from a referral MS research center database. Furthermore, the association of the T1 hypointense lesions would be sought with the disability of the patients calculated by the Expanded Disability Status Scale (EDSS).

## Materials and methods

### Study population

The list of patients with the final diagnosis of NMOSD based on 2015 NMOSD IPND criteria [1] was collected from the MS research center of Sina Hospital (Tehran University of Medical Sciences, Tehran, Iran). Patients with less than 6-month intervals from their last clinical attack were excluded. The range of interval between MRIs and the last relapse was from 6 months to two years. Their demographic data, disease-related information (onset age, disease duration, and EDSS), and lab data (i.e., anti-aquaporin 4; AQP-4) were extracted from the archived files of the inpatients and the outpatients’ units. Fixed Cell Base Assay (CBA) test was performed to identify AQP-4 Ab in serum in our study. The current study is approved by the research and ethics committee of Sina Hospital MS research center. The patients were well informed at the time of their admission/present for the future use of their innominate data.

### Images analysis

The last brain and spine MRIs were reviewed by two radiologists. All MRI acquisitions were done by 1.5 Tesla scanners. A data set was complete comprising the following data according to the consensus of the two radiologists: Presence of T1 hypointense lesions in the hemispheric white matter, corticospinal tract, peri ependymal regions around the 3rd and 4th ventricles/aqua duct, periependymal regions around the 1st and 2nd ventricles/ corpus callosum, superior tegmentum, and other locations comprising basal ganglia, cerebellum, and cortical/subcortical regions. The presence of T1 hypointense lesions in the spinal cord was also registered.

### Theory/Calculation

BH was defined as lesions with high signal intensity on T2 and low signal intensity on T1 sequences. It is worth noting that those lesions that revealed contrast enhancement after Gadolinium administration were not counted as T1-hypointense lesions. Furthermore, T2/FLAIR hyperintense brain and cord lesions that had high T2 and normal T1 intensities were documented in this study. The number, distribution, and extent of the signal intensities of the T1 hypointense lesions were gathered. The length of the spinal cord lesions was measured by counting the number of vertebral bodies leveling up all cord lesions.

We divided cases into two groups: AQP-4 positive and AQP-4 negative. Data were analyzed with SPSS v23. Independent sample T-test and ANOVA were employed to compare the score of EDSS in different categories (i.e., the presence of T1 hypointense lesions in general and different locations and 3 different CSF-like, cortex-like, and intermediate T1 signal intensities). Correlation of the presence/absence of the T1 hypointense lesions and AQP-4 were assessed by Chi-square test. A general linear model was designed to study the effect of the length of the spinal lesions on the association of T1 hypointense lesions and EDSS.

## Results

### General features

Eighty-three patients were enrolled with NMOSD presented at the MS research center of Sina Hospital from March 2006 to March 2021. Ten patients were excluded due to restricted access to their brain, spinal MRI, or critical clinical data. Final analyses were performed on 73 patients with a mean ± SD age of 28.0 ± 10.4 years at the onset of the symptoms and 33.4 ± 11.3 years at the time of EDSS calculation. The mean ± SD disease duration (i.e., symptoms onset to EDSS calculation) was measured at 5.3 ± 4.6 years. AQP-4 was positive in 36 (49.3%) patients. The mean ± SD of the EDSS score was 3.0 ± 1.7. The basic characteristics of the 73 patients are presented in Table 1.

**Table 1:** Demographic Characteristics of the Subjects

Variables	NMOSD^a^ with Positive AQP4^a^ 36 (49.3)	NMOSD with Negative AQP4 37 (50.7)
Age (years)	34.7 ± 12.2	32.0 ± 10.3
Gender		
Female	30 (83.3)	28 (75.7)
Male	6 (16.7)	9 (24.3)
Age at onset (years)	30.5 ± 12.1	25.6 ± 7.8
Minimum, Maximum age at onset (years)	12, 64	16, 45
Disease duration (years)	4.3 ± 3.1	6.3 ± 5.6
EDSS	3.2 ± 1.7	2.7 ± 1.6

^a^Quantitative variables are presented as mean ± SD, whereas qualitative variables are reported as number (%)

*EDSS* Expanded Disability Status Scale, *AQP-4* Aquaporin-4, *NMOSD* Neuromyelitis Optica Spectrum Disorder

### Description of the brain T1 hypointense lesions

**Fig. 1:**
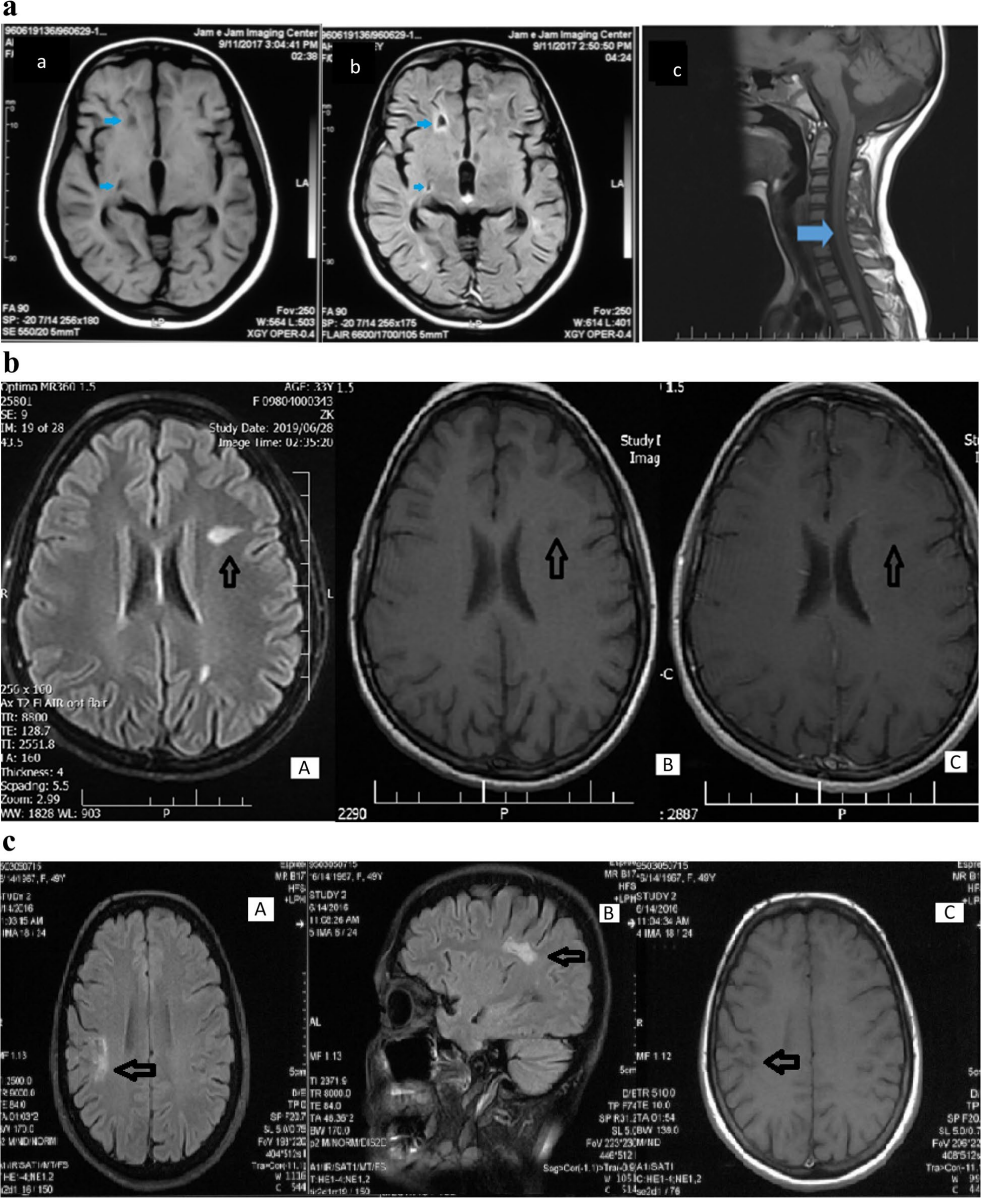
**A** Example of T1 Hypointense lesions in a 23-year-old female with NMOSD and positive AQP-4 test. The visible T1 hypointense lesions are depicted in the white matter on T1 (a) and corresponding FLAIR(b) images located in the hemispheric and periventricular white matter. No enhancement was noted in T1 enhanced images (not shown in the Figure). Also, the centrally-located long T1 hypointense lesion is shown in cervical cord (c). **B** Hemispheric white matter T1 hypointense lesions (black arrows) in a 33-year-old female; The most prevalent location of T1 hypointense lesions in chronic phases of Neuromyelitis Optica. **A** FLAIR axial sequence. **B** T1-weighted axial sequence. **C** T1-weighted post contrast sequence; No obvious enhancement after contrast injection appears in the lesion. **C** Hemispheric white matter T1 hypointense lesion (black arrows); **A** FLAIR axial sequence. **B** FLAIR sagittal sequence. **C** T1-weighted axial sequence

The total number of T1 hypointense lesions was 63. The most common location was the hemispheric white matter (*n* = 39; 7 out of 39 were ≥ 2 cm) followed by the peri ependymal regions around the 1st and 2nd ventricles/ corpus callosum (*n* = 16; 3 out of 16 were ≥ 2 cm) (Table 2). We did not detect any same lesions in the superior tegmentum (Fig. 1).

**Table 2:** Distribution of T1 Hypointense lesions in brain

		**Number (%)**		
**Distribution**	**Size**	**AQP-4 positive**	**AQP-4 negative**	**Total**
**Hemispheric white matter**	< 2cm	16 (25.4)	17 (27.0)	33(52.4)
	> 2cm	3(4.8)	4(6.3)	7(11.1)
**Corticospinal tracts**	< 2cm	1(1.6)	2(3.1)	3(4.8)
	> 2cm	0	0	0
**Periependymal around 3rd and 4th ventricles/aqua duct**	< 2cm	2(3.1)	1(1.6)	3(4.8)
	> 2cm	0	0	0
**Periependymal regions around 1st and 2nd ventricles/ corpus callosum**	< 2cm	6(9.5)	7(11.1)	13(20.6)
	> 2cm	0	3(4.8)	3(4.8)
**Superior tegmentum**	< 2cm	0	0	0
	> 2cm	0	0	0
**Others**^**a**^	< 2cm	0	1(1.6)	1(1.6)
	> 2cm	0	0	0

Total number of T1 hypointense lesions = 63

^a^basal ganglia, cerebellum and cortical/subcortical lesions

Ten patients (15.9%) showed CSF-like signal intensity in T1 hypointense lesions (as a surrogate for cystic changes) in 16 lesions. This pattern was particularly observed most commonly in the hemispheric white matter lesions (56%).

### Imaging characteristics and EDSS

The characteristics of patients with corresponding EDSS calculations are presented in Table 3. Out of all patients, 22 (30.1%) had T1 hypointense brain lesions, 46 (63.0%) T2/FLAIR hyperintense lesions, 18 (24.7%) cord T1 hypointense lesions, and 69 (94.5%) T2/FLAIR cord lesions.

**Table 3:** Correlation of MRI features with EDSS in NMOSD subjects

		**AQP-4 negative**				**AQP-4 positive**				**Total**			
		**Number of BH**	**Mean EDSS**	**SD**	**Sig**	**Number of BH**	**Mean EDSS**	**SD**	**Sig**	**Number of BH**	**Mean EDSS**	**SD**	**Sig**
**Brain T1 hypointense lesion**	No	25	2.6	1.7	0.522	26	2.8	1.7	0.005	51	2.7	1.7	0.022
	Yes	12	3.0	1.5		10	4.5	1.1		22	3.7	1.5	
**Hemispheric white matter T1 hypointense lesion**	None	28	2.4	1.6	0.101	25	2.9	1.7	0.888	53	2.7	1.7	0.014
	< 2 cm	9	2.8	1.6		4	4.4	1.4		13	3.3	1.7	
	= > 2 cm	3	4.7	1.1		4	4.5	0.9		7	4.6	0.9	
**Corticospinal tract T1 hypointense lesion**	No	36	2.6	1.6	0.051	34	3.2	1.7	0.680	70	2.9	1.7	0.112
	Yes	1	6.0			2	3.7	1.8		3	4.5	1.8	
**Periependymal (Lateral Ventricles) T1 hypointense lesion**	No	34	2.7	1.6	0.342	39	3.2	1.7	0.875	73	3.0	1.7	0.915
	Yes	0	-			0	-			0	-		
**Periependymal (3rd and 4th ventricles) T1 hypointense lesion**	No	34	3.0	1.4	0.065	36	2.8	1.8	0.574	70	2.9	1.7	0.216
	Yes	1	5.0			2	3.7	1.8		3	4.2	2	
**Cord T1 hypointense lesion**	No	28	2.7	1.7	0.022	27	2.8	1.7	0.121	55	2.7	1.7	0.055
	Yes	8	2.7	1.7		10	4.6	0.9		18	3.6	1.6	
**Brain T2/FLAIR hyperintense lesions**	No	12	2.2	1.8	0.212	15	2.4	1.6	0.014	27	2.3	1.7	0.013
	Yes	25	2.9	1.6		21	3.8	1.6		46	3.3	1.6	
**Cord T2/FLAIR hyperintense lesions**	No	1	5.0		0.162	3	2.0	1.0	0.194	4	2.7	1.7	0.790
	Yes	36	2.6	1.6		33	3.4	1.7		69	3.0	1.7	

Significance (sig.) level of 0.05 is reported for T test and ANOVA for comparisons of EDSS between variables with 2 and 3 categories

*EDSS* Expanded Disability Status Scale, *SD* Standard Deviation

**Fig. 2:**
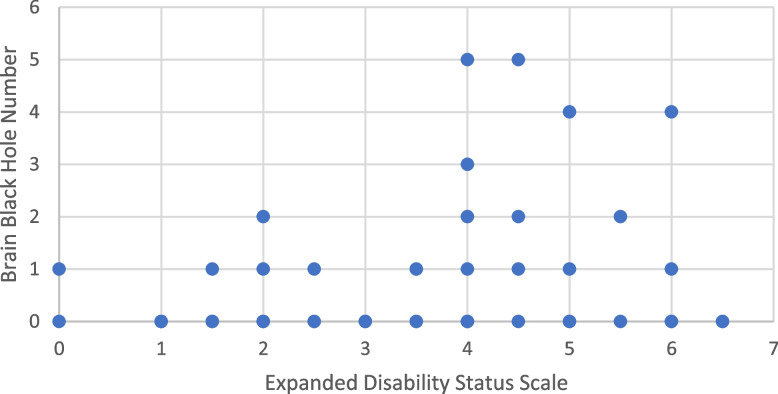
The correlation of number of T1 hypointense lesions with Expanded Disability Status Scale (EDSS). All patients with > 4 T1 hypointense lesions had EDSS > 4 and 11 out of 13 patients with > 2 T1 hypointense lesions had EDSS > 3

Figure 2 shows that EDSS was of higher values in patients with T1 hypointense brain lesions (2.7 ± 1.7 vs. 3.7 ± 1.5; *p* = 0.02) so that all patients with more than four T1 hypointense brain lesions had EDSS higher than 4 (7 patients) and 11 out of 13 patients (84.6%) with the number of two T1 hypointense brain lesions had EDSS > 3 indicating non-mild disability. EDSS was higher in the patients with T2/FLAIR hyperintense brain lesions (3.6 ± 1.6 vs 2.3 ± 1.7; *p* = 0.01). When splitting cases into AQP-4 positive and negative, a significant correlation between higher EDSS and the presence of brain T1 hypointense lesions and brain T2/FLAIR hyperintense lesions were present in AQP-4 positive cases, unlike AQP-4 negatives (*p*-value = 0.005 for T1 hypointense lesions and *p*-value = 0.014 for T2/FLAIR hyperintense lesions) (Table 4). Also, no associations existed between the extent of the signal intensity of T1 hypointense brain lesions with EDSS (*p* = 0.46).

**Table 4:** Independent Samples Test for correlation of Brain T1 hypointense and T2/FLAIR hyperintense lesions with EDSS

				**Levene's Test for Equality of Variances**		**t-test for Equality of Means**				
				**F**	**Sig**	**t**	**df**	**Sig. (2-tailed)**	**Mean Difference**	**Std. Error Difference**
**Brain T1 Hypointense Lesion**	Negative AQP-4 Ab	EDSS	Equal variances assumed	0.95	0.335	-0.64	35	0.522	-0.37	0.58
			Equal variances not assumed			-0.68	25.61	0.497	-0.37	0.54
	Positive AQP-4 Ab	EDSS	Equal variances assumed	2.38	0.132	-2.98	34	0.005	-1.73	0.58
			Equal variances not assumed			-3.51	23.92	0.002	-1.73	0.49
**Brain T2/FLAIR Hyperintense Lesions**	Negative AQP-4 Ab	EDSS	Equal variances assumed	0.00	0.964	-1.27	35	0.212	-0.73	0.57
			Equal variances not assumed			-1.21	19.34	0.240	-0.73	0.60
	Positive AQP-4 Ab	EDSS	Equal variances assumed	0.04	0.833	-2.58	34	0.014	-1.40	0.54
			Equal variances not assumed			-2.58	30.28	0.015	-1.40	0.54

Significance (sig.) level of 0.05 is reported for T test

*EDSS* Expanded Disability Status Scale

Cord T1 hypointense lesion was presented in 18 (24.6%) patients with marginally higher EDSS values (2.7 ± 1.7 vs. 3.6 ± 1.6; *p* = 0.05). EDSS was almost similar between patients with and without the spinal cord lesions (non-T1 hypointense lesions) but correlated with the length of the spinal cord T2/FLAIR hyperintense lesions (*r* = 0.36; *p* = 0.02) and particularly with the length of the lesions of the thoracic spinal cord (*r* = 0.49; *p* < 0.001).

Moreover, no correlation existed between the length of cord lesions and the presence of spinal T1 hypointense lesions (*p* = 0.12).

EDSS was similar between positive and negative AQP-4 (2.7 ± 1.6 vs. 3.2 ± 1.7; *p* = 0.17). Also, a positive APQ-4 state was not more prevalent in patients with T1 hypointense brain lesions than those without (50.9 vs 45.4%; *p* = 0.80).

## Discussion

Several investigations have demonstrated that brain involvement is a relatively common phenomenon in NMOSD [14, 15]; however, few studies addressed the brain T1 hypointense lesions in this disorder [16]. Our results showed that 30.1% of NMOSD subjects developed brain T1 hypointense lesions. Furthermore, we showed that T1 hypointense lesions in the brain and the cord and T2/FLAIR hyperintense brain lesions present more in patients with higher EDSS and more corresponding disabilities. Additionally, after splitting patients into two groups, respecting their AQP-4 antibody status, we found that the presence of T1 hypointense lesions is correlated with higher EDSS only in AQP-4 positive cases, and no correlation existed between the presence of T1 hypointense lesions and EDSS in AQP-4 negatives. Likewise, the same results were acquired for T2/FLAIR hyperintense brain lesions. The reason might be different due to their distinct pathophysiology [17], which might potentially lead to different outcomes and consequences, one of which can be proposed as more tissue destruction known as black holes led to higher scores of EDSS and severe disease in AQP-4 positive cases, as our results suggested. To the best of our knowledge, this entity has not previously been mentioned. Also, it should be mentioned that in our study, no significant correlation between EDSS and T1 hypointense lesions in each separate location was detected, neither in AQP-4 negative nor AQP-4 positive cases. This is probably due to the small sample size in each group above. Hence, studies with larger sample sizes can better elucidate this potential correlation.

Although the presence of T2 hyperintense spinal cord lesions did not correlate with EDSS, the spinal cord lesion length, specifically in the thoracic cord, correlated with higher EDSS, consequently, even though the spinal cord lesions are of great importance for the diagnosis of NMOSD, brain lesions (either T1 hypointense lesions or T2/FLAIR hyperintense lesions), cord T1 hypointense lesions, and more extensive T2/FLAIR hyperintense cord lesions predict more disabilities.

There is a substantial body of evidence showing that the progression of brain T1 hypointense lesions is a sign of permanent demyelination and axonal impairment in MS. This damage is believed to be caused by tissue damage or increased water influx through an impaired blood–brain barrier. Additionally, there was a trend toward an association between hypointense lesions and a total portion of matrix destruction [18]. The similar hypothesis can be proposed as the mechanism of neurological deficit occurs while NMOSD T1 hypointense lesions evolve.

The correlation between the existence of T1 hypointense lesions in MS and clinical outcome and disability is not yet well-elucidated and was controversial in different studies [19]. The relevance of brain T1 hypointense lesions and EDSS has not been previously explained in patients with NMOSD. As mentioned before, EDSS scores were significantly higher in patients with brain T1 hypointense lesions than patients without such lesions. Additionally, for the distribution of the brain T1 hypointense lesions, the most common location for these lesions was hemispheric white matter, followed by peri ependymal regions. The presence of brain T1 hypointense lesions in hemispheric white matter was correlated with significantly higher disability scores. In the study conducted by Kim et al. on 63 patients with NMOSD, the most frequently cerebral region revealing T1 hypointense lesions was the corpus callosum, followed by the corticospinal tract and cerebral white matter, which is inconsistent with our finding [16]. Among brain lesions they detected in the patients, 18% showed focal cystic changes, which is relatively similar to our results (15.9%). However, in the current study, the most common location for the CSF-like T1 Hypointense lesions was the hemispheric white matter. At the same time, cystic changes were observed most commonly in the corticospinal tract in the report above. We did not find any relationship between the signal intensity pattern in T1 hypointense brain lesions and EDSS. In that study by Kim et al., the impact of cystic changes on motor disability was not fully evaluated [16]. It should be considered that patients in the current study had an attack-free interval of 6 months and were possibly in remission. The difference in the distribution of the lesions may relate to the possible different populations studied by Kim et al. during the acute/chronic phases and the current study during remission. Our observation, that brain T1 hypointense lesions were associated with higher EDSS scores and subsequent poor clinical outcomes, suggests that the underlying pathological lesion is probably the result of tissue destruction rather than edema, to say permanent rather than transient.

There is compelling evidence about the influential role of cord involvement in NMOSD diagnosis [20], although its effect on the disability remains controversial [21, 22]. The results indicated that T2/FLAIR hyperintense cord lesion length, especially in the thoracic region, was correlated with a higher EDSS score, but the presence of T1 hypointense lesions or that T2/FLAIR hyperintense lesions had no significant correlation with it. As so, the extent of the cord involvement is more important than the presence of the lesions. Cord lesion length has been previously shown to correlate with the nadir and residual EDSS in a study by Bonnan et al. on 69 Afro-Caribbean NMOSD patients [23]. Furthermore, another previous investigation revealed an additive number of cord segments at the remission, and the presence of T1 hypointense cord lesion at myelitis attack were related to higher disabilities in a large multicenter cohort study [24]. Based on these findings, the length of the cord lesion could be a surrogate marker of potentially severe outcomes that may amend treatment intensification.

In previous investigations, the presence of AQP-4 showed a potential prognostic significance, which is inconsistent with the result of the current study [25]. In the same way, we did not find any relationships between AQP-4 positivity with the presence of brain T1 hypointense lesions. Nonetheless, as previously mentioned, we recognized that the presence of T1 hypointense brain lesions correlates with higher EDSS in AQP-4 positive cases. In 2021, Hsu et al. worked on correlations among disability, anti-AQP4 antibody status, and prognosis in patients with NMOSD. The topographic distributions of the spinal cord lesions might relate to different serum anti-AQP4 antibody statuses in their investigation [26]. Of concern, they did not evaluate the impact of brain lesions on EDSS scores.

We acknowledge the limitations of the current study. First, given the low prevalence of NMO, the number of patients was relatively small and thus may have been underpowered to evaluate any MRI predictive variables on disability. However, the sample size of the current study is one of the largest reported samples of patients with NMO. Second, as this was a retrospective study, no standardized imaging protocol was used. Thus some data were heterogeneous in scanners and protocols, with subsequent problems with direct comparison across patients. More studies with more cases, especially multi-center ones, will shed more light on this aspect of NMOSD imaging. This study may help clinicians consider BH as one of the imaging surrogate markers in predicting disability and treatment selection. Third, our study has been performed on stable patients referred to outpatient’s clinics of Sina Hospital, a referable center for NMOSD. Some of our patients had their first relapses and their management in other cities and centers and were then referred to our center after being stable. So, we did not have access to their total relapse number, and detailed information about annualized relapse rate.

## Conclusion

In the current study, 30.1% of NMOSD subjects developed brain T1 hypointense lesions, and the presence of T1 hypointense brain lesions correlates with higher EDSS in AQP-4 positive patients with NMOSD. Moreover, the presence of T2 hyperintense spinal cord lesions did not correlate with EDSS, though the spinal cord lesion length, more specifically in the thoracic cord, correlated with higher EDSS.

## Data Availability

The datasets used and/or analyzed during the current study are available from the corresponding author on reasonable request.

## Abbreviations

NMOSD: Neuromyelitis Optica Spectrum Disorder
EDSS: Expanded Disability Status Scale
AQP-4: Aquaporin-4
MRI: Magnetic Resonance Imaging
BH: Black Holes
MS: Multiple Sclerosis
CSF: Cerebrospinal Fluid
